# Bilateral Keratoconus in a Patient with Isolated Foveal Hypoplasia

**DOI:** 10.18502/jovr.v15i2.6745

**Published:** 2020-04-06

**Authors:** Kiana Hassanpour, Ramin Nourinia, Nazanin Behnaz, Mohsen Azarmina, Setareh Jalali, Danial Roshandel

**Affiliations:** ^1^ Ophthalmic Research Center, Shahid Beheshti University of Medical Sciences, Tehran, Iran

##  PRESENTATION

We present a 29-year-old woman complaining of low visual acuity since childhood being deteriorated in the past six months.

Ophthalmic examination revealed low-amplitude, jerky horizontal nystagmus in both eyes that worsened in end-gaze. The manifest refraction was –3.00 –9.5 × 20° and –2.5 –9.00 × 150° in her right and left eyes, respectively. The best spectacle-corrected visual acuity (BSCVA) was 20/70 in both eyes. In both eyes, BCVA with rigid gas permeable lenses was 20/50. Direct and consensual pupillary light reflexes were within normal limits and no relative afferent pupillary defect was detected. Color vision tested by Ishihara's color plates revealed no deficits. Slit lamp examination showed mild paracentral corneal thinning and bulging, Fleischer's ring, and Vogt's striae in both eyes. Iris examination revealed no sign of transillumination. Intraocular pressure measured by Goldmann applanation tonometry was 15 mmHg in both eyes. Dilated fundus examination revealed generalized chorioretinal atrophy and no foveal reflex and no macular yellow hue. Retinal capillaries at the macular area showed mild irregularity. Both optic discs were pink and had sharp margins.

**Figure 1:**
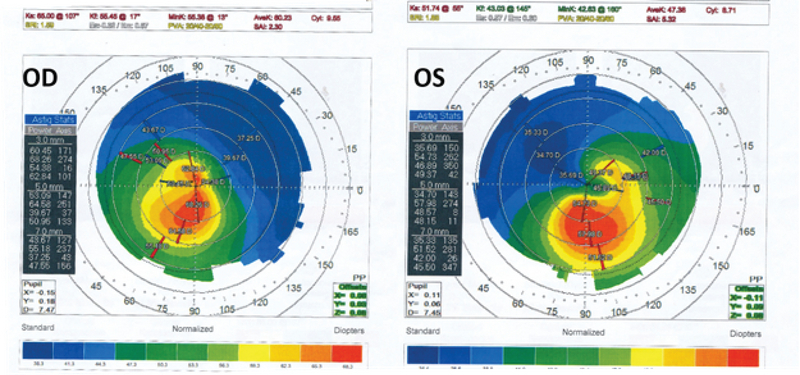
Placido disk-based topography showing asymmetric bow-tie patterns with skewed radial axes (SRAX) in both eyes. The steep and flat keratometry of the right eye was 65.00 D at 107º and 55.17 D at 17º, respectively. In the left eye, the steep and flat keratometry was 51.74 D at 55º and 43.03 D at 145º, respectively.

**Figure 2:**
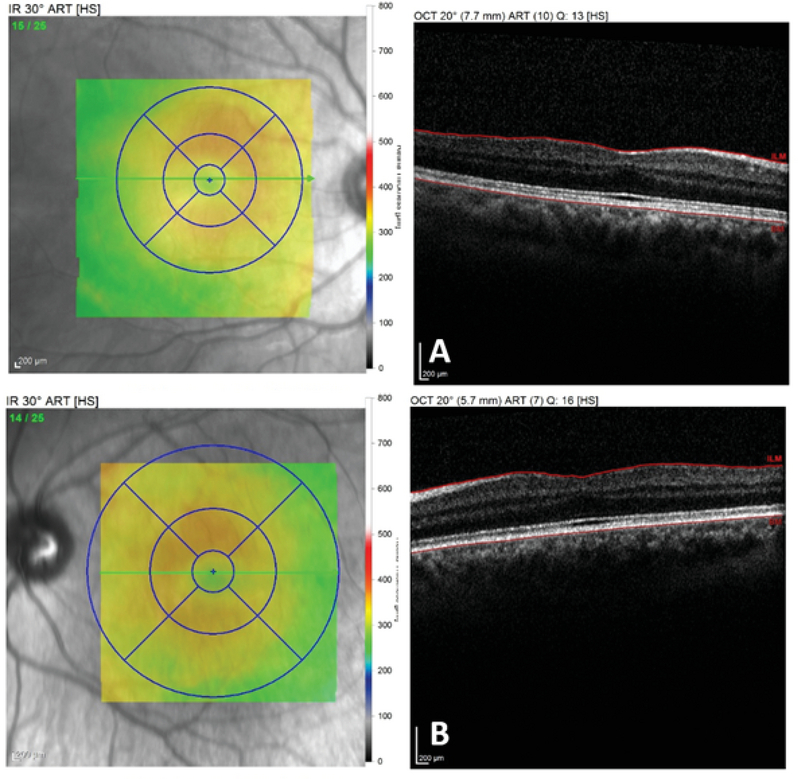
Spectral-domain optical coherence tomographic section through the center of the macular area of the right (A) and left (B) eyes. A shallow foveal pit, continuity of the inner retinal layers, the presence of outer nuclear layer (ONL) widening, and outer segment (OS) lengthening in the macula of both eyes are noted.

**Figure 3:**
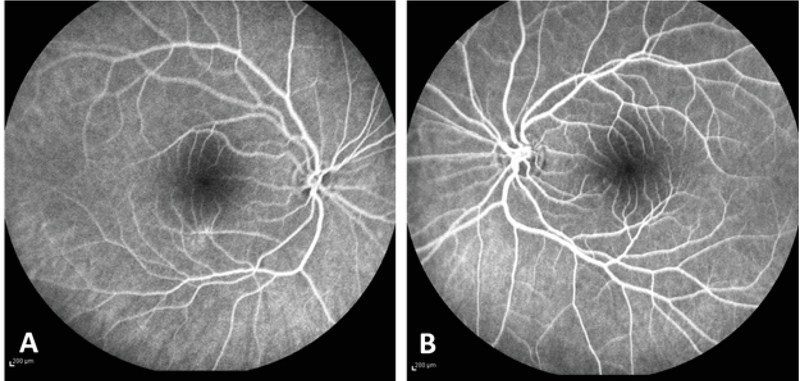
Fluorescein angiography of the right (A) and left (B) eyes showing a small capillary-free zone in each eye, with almost normal masked fluorescence in the macular area due to the presence of the macular pigments.

Placido disk-based topography showed asymmetric bow-tie pattern with skewed radial axes in both eyes [Figure 1]. Spectral domain optical coherence tomography (OCT) scans showed the absence of the foveal depression and persistent inner retinal layers (similar to paramacular scans) within the fovea in both eyes [Figure 2].

Fluorescein angiography (FA) revealed a small capillary-free zone in both eyes with almost normal masked fluorescence in the macular area due to the presence of macular pigments [Figure 3].

Electroretinograms (both photopic and scotopic) and electrooculograms showed normal results in both eyes.

##  DISCUSSION

Continuity of the inner retinal layers and absence or decrease of the foveal depression (also known as fovea plana) are typical OCT findings in foveal hypoplasia (FH).^[1]^ In our case, along with the

presence of the typical clinical findings of FH, macular OCT revealed a shallow foveal pit and abnormal persistence of the inner retinal layers in the foveal area. Diagnosis of FH was further supported by demonstrating the abnormal extension of the retinal capillaries toward the foveal area in FA.

Many reports have linked chronic eye rubbing and keratoconus, especially in children.^[2]^ Our patient also had a history of habitual eye rubbing since her childhood, which may explain the development of Keratoconus (KC). However, because of the consanguineous marriage of her parents, the inheritance of a recessive mutation should also be considered as a possible etiology for these findings. The visual system homeobox (*VSX1*) gene encodes VSX1, which is mainly present in human retinal cells. Interestingly, mutations in this multifunctional gene have been described in KC and posterior polymorphous corneal dystrophy and may result in abnormal development of both the cornea and the retina.^[3]^ Although this rare and unique association may be an incidental finding, comprehensive genetic study is needed to confirm this hypothesis.

##  Financial Support and Sponsorship

None.

##  Conflicts of Interest

There are no conflicts of interest.
